# Transcriptome Analysis Showed a Differential Signature between Invasive and Non-invasive Corticotrophinomas

**DOI:** 10.3389/fendo.2017.00055

**Published:** 2017-03-22

**Authors:** Leonardo Jose Tadeu de Araújo, Antonio Marcondes Lerario, Margaret de Castro, Clarissa Silva Martins, Marcello Delano Bronstein, Marcio Carlos Machado, Ericka Barbosa Trarbach, Maria Candida Barisson Villares Fragoso

**Affiliations:** ^1^Laboratory of Hormones and Molecular Genetics LIM-42, University of São Paulo Medical School, São Paulo, Brazil; ^2^Laboratory of Quantitative Pathology, Center of Pathology, Adolfo Lutz Institute, São Paulo, Brazil; ^3^Division of Metabolism, Endocrinology and Diabetes, University of Michigan, Ann Arbor, MI, United States; ^4^Internal Medicine Department, Ribeirao Preto Medical School, University of São Paulo, Ribeirao Preto, Brazil; ^5^Neuroendocrine Unit, Division of Endocrinology and Metabolism, University of São Paulo Medical School, São Paulo, Brazil; ^6^Endocrinology Service, AC Cancer Center, São Paulo, Brazil; ^7^Laboratory of Cellular and Molecular Endocrinology LIM-25, University of São Paulo Medical School, São Paulo, Brazil

**Keywords:** Cushing’s disease, gene expression, neuroendocrine tumors, microarray, anterior pituitary

## Abstract

ACTH-dependent hypercortisolism caused by a pituitary adenoma [Cushing’s disease (CD)] is the most common cause of endogenous Cushing’s syndrome. CD is often associated with several morbidities, including hypertension, diabetes, osteoporosis/bone fractures, secondary infections, and increased cardiovascular mortality. While the majority (≈80%) of the corticotrophinomas visible on pituitary magnetic resonance imaging are microadenomas (MICs, <10 mm of diameter), some tumors are macroadenomas (MACs, ≥10 mm) with increased growth potential and invasiveness, exceptionally exhibiting malignant demeanor. In addition, larger and invasive MACs are associated with a significant increased risk of local complications, such as hypopituitarism and visual defects. Given the clinical and molecular heterogeneity of corticotrophinomas, the aim of this study was to investigate the pattern of genetic differential expression between MIC and MAC, including the invasiveness grade as a criterion for categorizing these tumors. In this study, were included tumor samples from patients with clinical, laboratorial, radiological, and histopathological diagnosis of hypercortisolism due to an ACTH-producing pituitary adenoma. Differential gene expression was studied using an Affymetrix microarray platform in 12 corticotrophinomas, classified as non-invasive MIC (*n* = 4) and MAC (*n* = 5), and invasive MAC (*n* = 3), according to modified Hardy criteria. Somatic mutations in *USP8* were also investigated and mutations were identified in six cases. Differential expression analysis demonstrated that non-invasive MIC and MAC have a similar genetic signature, while invasive MACs exhibited a differential expression profile. Among the genes differentially expressed, we highlighted *CCND2, ZNF676, DAPK1*, and *TIMP2*, and their differential expression was validated through quantitative real-time PCR in another cohort of 15 non-invasive and 3 invasive cortocotrophinomas. We also identified potential biological pathways associated with growth and invasiveness, TGF-β and G protein signaling pathways, DNA damage response pathway, and pathways associated with focal adhesion. Our study revealed a differential pattern of genetic signature in a subgroup of MAC, supporting a genetic influence on corticotrophinomas in patients with CD.

## Introduction

ACTH-dependent hypercortisolism caused by a pituitary adenoma [Cushing’s disease (CD)] is the most common cause of endogenous Cushing’s syndrome, representing ≈10% of all pituitary adenomas (1, 2). CD is often associated with several morbidities, including hypertension, diabetes, osteoporosis/bone fractures, secondary infections, and increased cardiovascular mortality (3–5). The severity of the clinical manifestations varies according to the level of hormonal overproduction, exposure time, and glucocorticoid receptors sensitivity. While the majority of the corticotrophinomas visible on pituitary magnetic resonance imaging (MRI) are microadenomas (MICs, <10 mm of diameter), some macroadenomas (MACs, ≥10 mm) exhibit increased growth potential and invasiveness, exceptionally exhibiting malignant behaviour (6–9). In addition, larger and invasive MACs are associated with a significant increased risk of local complications, such as hypopituitarism and visual loss (6, 8, 10).

It has been reported that the persistence of cortisol response to desmopressin, in the early postoperative period, could help to identify CD patients with initial remission, who present risk for later recurrence (11). However, apart from clear radiological signs of invasiveness, strong predictors of poor surgical outcomes are not available and early predictors of tumor growth and invasiveness would be of clinical value.

Over the years, molecular markers have emerged as potential predictors of tumor aggressiveness. Previously, the overexpression of *fibroblast growth factor receptor-4 (FGFR4*) was correlated with the proliferation marker Ki-67 and tended (but not significantly) to be found in invasive pituitary adenomas (12). Also, we have suggested that increased *FGFR4* expression levels and the presence of homozygosis for the *FGFR4* Gly388 allele were associated with a higher frequency of postoperative recurrence and persistence of CD, respectively (13). Evidence suggests that the signaling properties of N-cadherin, with particular emphasis on its cross talk with cell surface partners such as FGFR4 and NCAM, are important in pituitary tumorigenesis (1, 7, 14, 15). The potential oncogenic contribution of fibroblast growth factors and their receptors to pituitary tumorigenesis and invasiveness is still unclear, although it is well established that these growth factors and respective receptors are important for a variety of biological processes, including mitogenesis, differentiation, development, angiogenesis, and tumorigenesis (16).

Recently, Reincke et al. (17) identified somatic heterozygous mutations in *USP8* (*ubiquitin-specific protease Y*), an important regulator of the epidermal growth factor receptor (EGFR) downstream signaling, in ≈36% of corticotrophinomas. Noteworthy, the majority of the cases with *USP8* mutations were MICs from young patients with CD (17, 18). The authors suggest that in the presence of such mutations, EGFR ubiquitination, and turnover would be impaired, causing its accumulation in the plasma membrane, where the receptor remains active and stimulating proopiomelanocortin transcription and ACTH secretion and also contributing to corticotrophic tumorigenesis (17). However, in these studies, the authors did not study any invasive corticotroph tumor.

Lately, a review has collected data from studies of gene and protein expression in corticotrophinomas, compared to normal pituitary gland, with the aim of prioritizing targets that could contribute to the improvement of the molecular diagnosis of CD. Among the differentially expressed genes and respective proteins in corticotrophinomas, the most well-established candidates, emphasized in multiple studies, were *NEUROD1* (neuroD1), *h*P*TTG1* (securin), *HSD11B2* (11β-hydroxysteroid dehydrogenase 2), *AKT* (Akt protein kinase B), *CCND1* (cyclin D1) (overexpressed), *CDKN1B* (p27^Kip1^), *CDKN2A* (p16), *KISS1* (kisspeptin), and *ACTHR* (ACTH-R) (underexpressed) (19).

*h*P*TTG1* is a member of the securin family, which regulates sister chromatid separation during mitosis. Evidence suggests tissue-specific expression of three *hPTTG1* genes and potential roles for each of them in tumorigenesis, cell transformation, DNA repair, angiogenesis, and gene regulation (20). It is noteworthy that it has already been demonstrated that increased *hPTTG1* expression was associated with invasiveness in functional pituitary adenomas (21). This overexpression was not only observed in pituitary adenomas but also in various non-pituitary and pituitary carcinoma, at even higher levels (21, 22), and these data contributed to *hPTTG1* being classified as a proto-oncogene. Years later, Filippella et al. (23) demonstrated a positive correlation between *hPTTG1* expression and the Ki-67 nuclear proliferation index, with the expression, aggressiveness, invasiveness, and recurrence potential of pituitary adenomas.

According to the clinical and molecular heterogeneity of corticotrophinomas, we aimed to explore the pattern of gene expression associated with tumor growth and invasiveness. Therefore, we studied a cohort of corticotrophinomas, with different phenotypic features, by microarray analysis.

## Subjects and Methods

### Patients and Tumor Samples

Fifteen subjects (14 females and 1 male—age ranged between 14–70 years) with clinical, laboratorial, radiological, and histopathological diagnosis of ACTH-producing pituitary adenoma were included in this study.

Diagnosis of ACTH-dependent Cushing’s syndrome was based on typical clinical features and standard hormonal criteria: increased 24-h urinary cortisol excretion, loss of circadian rhythm of cortisol secretion (increase of nocturnal serum cortisol and/or nocturnal salivary cortisol), lack of suppression of serum cortisol after a low-dose dexamethasone test (1 mg orally overnight), and elevated or inappropriate normal plasma ACTH levels (>15 pg/mL) (24, 25).

Ethical approval was obtained from the institutional review boards of Institution (Comissao de Etica para Analise de Projetos de Pesquisa—CAPPesq), and informed consent was obtained from all participants in written form and in accordance with the Declaration of Helsinki.

Tumoral tissue specimens were obtained during transsphenoidal surgery. Tissue portions not used for histology and were snap-frozen in liquid nitrogen, preceding RNA/DNA extraction (AllPrep DNA/RNA kit^®^—Qiagen GmbH, Hilden, Germany). After extraction, quality (A260/A280 A260/A280 acceptable ratio range of 1.8–2.0) and integrity were assessed by absorbance measures in a NanoDrop™ spectrophotometer (Thermo Scientific) and agarose gel electrophoresis.

### Tumor Characterization

Corticotroph tumors were characterized by immunostaining for ACTH. Tumor size and invasiveness were defined based on preoperative pituitary MRI and perioperative findings (26). We adopted the modified Hardy, as follows: grade I, enclosed MIC (tumor <10 mm); grade II, enclosed MAC (tumor ≥10 mm); grade III, localized perforation of the sellar floor; and grade IV, diffuse destruction of the sellar floor (6, 27). Grade III and IV adenomas were considered invasive, and tumor invasion was based on the evidence of bone destruction and/or tumor extension within sphenoid and/or cavernous sinuses and/or brain, as confirmed at surgery (28, 29). In our cohort, the immunohistochemical markers for aggressiveness: elevated Ki-67 (>3%) and increased nuclear reaction for the p53 protein were not observed in both non-invasive and invasive group of corticotrophinomas that underwent immunohistochemistry analysis (Table 1).

**Table 1:** **Patients with Cushing’s disease included to microarray study**.

ID	Gender	Age (years)	Grade	Size (mm)	UC 50–310 μg/24 h	ACTH < 46 pg/mL	Invasion	Remission	Ki-67	p53
**Non-invasive microadenomas**										
1	F	39	I	6	610	38	Absent	No	2%	1%
2	F	41	I	6	572	39	Absent	No	2%	1%
3	F	32	I	7	388	54	Absent	Yes	NA	NA
4	F	39	I	8	961	63	Absent	Yes	NA	NA
**Non-invasive MACs**										
5	F	70	II	15	269	46	Absent	No	1%	1%
6	F	36	II	12	1,390	53	Absent	Yes	NA	NA
7	F	28	II	20	326	26	Absent	Yes	2%	NA
8	F	47	II	11	925	79	Absent	Yes	1%	1%
9	F	14	II	19	1,207	68	Absent	Yes	2%	NA
**Invasive MACs**										
10	F	43	III	25	445	150	RCS	No	1%	1%
11	F	30	IV	40	378	46	LCS	No	1%	2%
12	F	50	IV	18	395	111	RCS	No	1%	1%

*Non-invasive adenomas were classified according to Hardy (6) modified by Wilson (27), and invasive MACs were classified according to Knosp et al. (28). Total urinary cortisol was measured without extraction. The mean follow-up was 24 months. ID, patient number; UC, urinary cortisol, mean of three or more samples; NA, data not available; RCS, right cavernous sinus; LCS, left cavernous sinus; MACs, macroadenomas*.

We assessed the degree of contamination with normal pituitary tissue by measuring the expression levels of *POU1F1* and *TPIT* (the genes encoding the transcription factors Pit-1 and T-pit) as previously described by our group (13). Corticotrophinomas should exhibit high levels of *TPIT* expression and undetectable levels of *POU1F1*. On the other hand, *POU1F1* expression levels are significantly higher in the normal pituitary, since it is expressed by all the pituitary cell lineages, except the corticotrophic. After PCR analysis, 3 of our 15 primary samples were excluded, due to visible *POU1F1* expression, indicating possible contamination with normal pituitary tissue (Figure S1 in Supplementary Material).

Demographic and clinical characteristics of the remaining participants are summarized in Table 1.

### USP8 Analysis

As somatic mutations were recently described in the literature (17), we also performed a mutational analysis of the ubiquitin-specific protease 8 (*USP8*; Ensembl: ENSG00000138592) to investigate its presence/incidence in our cohort. It was accomplished using PCR amplification by specific primers (Table S1 in Supplementaary Material) and automatic SANGER sequencing according to Perez-Rivas et al. (18) in DNA extracted of patients tumors.

### Microarray Analysis

We extracted total RNA from four MICs (mean tumor size 6.75 ± 0.96 mm), five MACs (mean tumor size 15.40 ± 4.04 mm), and three invasive corticotrophinomas (mean tumor size 27.67 ± 11.24 mm).

Microarray mRNA expression profiling was performed using the Affymetrix Human Exon 1.0 ST^®^ chip (Affymetrix, Inc., Santa Clara, CA, USA). The mRNA was amplified into single-stranded-cDNA, fragmented, biotin-labeled, and hybridized to a chip using the Gene Chip^®^ WT Plus Reagent Kit (Affymetrix) according to the standard manufacturer’s protocols.

Raw microarray data were acquired using Affymetrix GeneChip operating software (GCOS) (Affymetrix) to yield CEL files. The success of hybridization was evaluated, and data were processed and analyzed using Affymetrix Expression Console^®^ 1.3 (Affymetrix) and gene level differential analysis workflow of Transcriptome Analysis Console^®^ 3.1 (Affymetrix). The background subtraction, normalization, and log base 2 transformation of gene signals were conducted using the robust multi-array average algorithm (30).

Differentially expressed genes were determined by comparing the groups MIC, MAC, and/or invasive using one-way ANOVA (*p*-value <0.05). Additionally, gene expression was compared by grouping tumors into non-invasive (*n* = 9) and invasive (*n* = 3). A Benjamini–Hochberg multiple testing correction adjusted *p*-value to smaller than 0.05, in addition a twofold change were used to select genes differentially expressed (31) (annotation file: HuEx-1_0-st-v2.na33.1.hg19.transcript.csv). Hierarchical clustering of the expression data was performed using the Euclidean distance metric and complete linkage method. Functional annotation was performed using DAVID^1^ and Enrich.^2^ Raw and normalized data of microarray analysis reported here were deposited in Gene Expression Omnibus database under accession number GSE72490.

### Quantitative Real-time PCR (qRT-PCR) Analysis

A subset of four target genes and was tested by qRT-PCR, and the assays are summarized in Table 2.

**Table 2:** **Characteristics of the gene probes used in quantitative real-time PCR**.

Gene symbol	Description	Assay
*TBP (housekeeping)*	TATA-box binding protein	4326322E
*CCND2*	Cyclin D2	HS00153380_M1
*ZNF676*	Zinc-finger 676 protein	HS00234278_M1
*DAPK1*	Death-associated protein kinase 1	HS05234480_M1
*TIMP2*	TIMP metalloproteinase inhibitor 2	HS01939480_S1

In order to perform data validation, we designed an additional cohort of 18 patients for this analysis; 5 patients from Neuroendocrinology Unit of Hospital das Clinicas of University of São Paulo Medical School and 13 from the Ribeirao Preto Medical School. These patients were selected and classified according to the same criteria described in the Section “Patients and Tumor Samples.” Demographic and clinical characteristics of the individuals, divided into invasive (*n* = 3) and non-invasive (*n* = 15) groups, are summarized In Table 3. Similar to our initial cohort, *USP8* mutations were also investigated in these patients.

**Table 3:** **Patients with Cushing’s disease included to the validation study using quantitative real-time PCR**.

ID	Gender	Age (years)	Grade	Size (mm)	NSC < 0.12 μg/dL	ACTH < 46 pg/mL	Invasion
**Non-invasive corticotrophinomas**							
2339	F	47	I	2	1.3	90.5	Absent
1138	F	54	I	3	1.6	53.3	Absent
2341	F	35	I	3	1.3	70.5	Absent
2337	F	27	I	5	2.0	95.2	Absent
2336	F	43	I	5	1.3	51.3	Absent
2332	F	23	I	5	1.6	35.9	Absent
2338	F	14	I	6	4.0	46.3	Absent
175	F	42	I	7	0.5	81.1	Absent
97	F	23	II	7	0.8	39.0	Absent
1132	F	45	II	10	2.3	73.9	Absent
2335	F	44	II	10	3.3	58.3	Absent
2330	F	47	II	12	17.8	128	Absent
1154	F	36	II	20	2.1	48.9	Absent
2331	F	31	II	20	1.9	118	Absent
1421	F	17	II	36	3.9	50.5	Absent
**Invasive corticotrophinomas**							
72	F	50	IV	18	0.08	66.0	RCS
*169*	F	40	III	25	0.08	83.6	RCS
*160*	F	59	IV	52	Not available	99.7	RCS

*Non-invasive adenomas were classified according to Hardy (6) modified by Wilson (27), and invasive macroadenomas were classified according to Knosp et al. (28)*.

*ID, patient number; NSC, nocturnal salivary cortisol; RCS, right cavernous sinus*.

Quantitative RT-PCR used TaqMan Universal PCR Master Mix and was run on an ABI 7000 Sequence Detection Systems^®^ (Applied Biosystems, Foster City, CA, USA). Each reaction was performed with 2.0 μL cDNA (obtained from 5 ng of total RNA). Each target gene was normalized to the Tata-box binding protein gene (*TBP)*, as previously standardized in our laboratory (32). Gene expressions values were calculated with the $2^{-\Delta \Delta C_{\text{t}}}$ method, where the Δ*C*_t_ value of the sample was determined by subtracting the average *C*_t_ value of the target gene from the average *C*_t_ value of the housekeeping gene. We used as a calibrator a commercial pool of normal pituitary gland (Pituitary Gland Human Poly A + RNA, Clontech, Japan).

## Results

Before microarray analysis, we performed the screening for mutations in *USP8* in our patients, and somatic variants were found in patients #2 and #5 (p.Ser718Pro), #4 (p.Ser718Cys), patients #3 and #6 (p.Pro720Arg), and #9 (p.Pro720Gln). Both mutations were found in heterozygosis and have been previously described (17, 18).

### Differentially Expressed Genes

Using Transcriptome Analysis Console (Affymetrix) software, a fold change and intensity-based filtering approach (>2.0-fold change and ANOVA *p* < 0.05) demonstrated only 48 transcripts differentially expressed in the MAC group compared to the MIC group: 41 transcripts were relatively overexpressed and 7 were underexpressed (Table 4). However, the hierarchical clustering analysis did not reveal differences that could successfully distinguish these groups (data not shown). In comparing corticotrophinomas grouped into invasive and non-invasive tumors, we observed 748 differentially expressed transcripts: 396 overexpressed and 352 underexpressed.

**Table 4:** **Differentially expressed genes in macro versus microcorticotrophinomas**.

Gene symbol	Fold change (linear)	Gene symbol	Fold change (linear)
*BMPR1B*	−4.92	*FLJ38379*	2.49
*KCNK1*	−3.66	*JUN*	2.52
*CCDC144A*	−2.43	*MLLT6*	2.58
*SV2B*	−2.39	*GDA*	2.63
*TLR6*	−2.11	*PDE4D*	2.67
*KLHL1*	−2.1	*FXYD1*	2.71
*CSH2*	−2.04	*BTBD11*	2.71
*C11orf63*	2.01	*FXYD3*	2.73
*LIN7A*	2.01	*UQCRFS1*	2.81
*DBN1*	2.05	*TGM2*	2.82
*PDGFC*	2.09	*STMN4*	2.88
*PDGFD*	2.16	*MT1G*	3.20
*KCNAB2*	2.21	*SLC7A5*	3.36
*DLG2*	2.21	*S1PR1*	3.37
*RPF2*	2.22	*CNR1*	3.41
*MC4R*	2.23	*TUBB2B*	3.47
*CTGF*	2.32	*MARCH1*	3.54
*ID 3402978*	2.32	*LYPD6B*	3.56
*SRP14*	2.33	*SCGB2A1*	3.69
*ID 2763154*	2.38	*ALDH1A1*	3.76
*PITPNM2*	2.40	*EFNA3*	3.81
*HLA-DOB*	2.40	*LOC100653008*	4.88
*CLVS1*	2.42	*ACSS3*	5.20
*ZNF208*	2.43	*TRPC7*	12.62

*ANOVA p-value <0.05; fold change cut off = 2; ID, transcript ID, no gene symbol available*.

**Figure 1:**
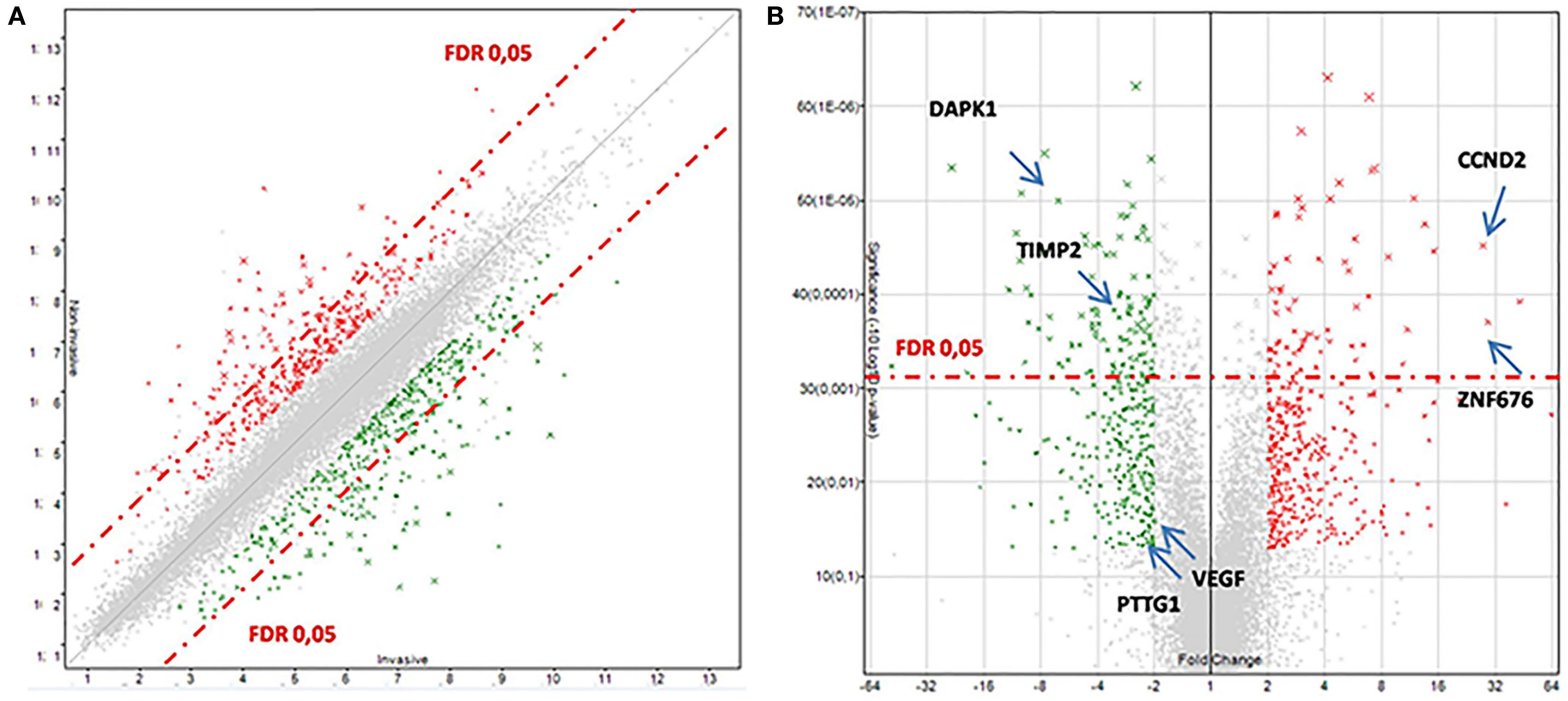
**Transcriptomic data between invasive and non-invasive corticotrophinomas**. **(A)** Scatter plot. **(B)** Volcano plot. The over- and underexpressed genes are represented in red and green, respectively. In these plots, genes with a fold change less than 2 and ANOVA *p*-value >0.05 are shown in light gray (in the center). The dashed red-line shows where false discovery rate (FDR) = 0.05, with points above the line having *p* < 0.05. Arrows highlights some genes discussed in the text.

**Figure 2:**
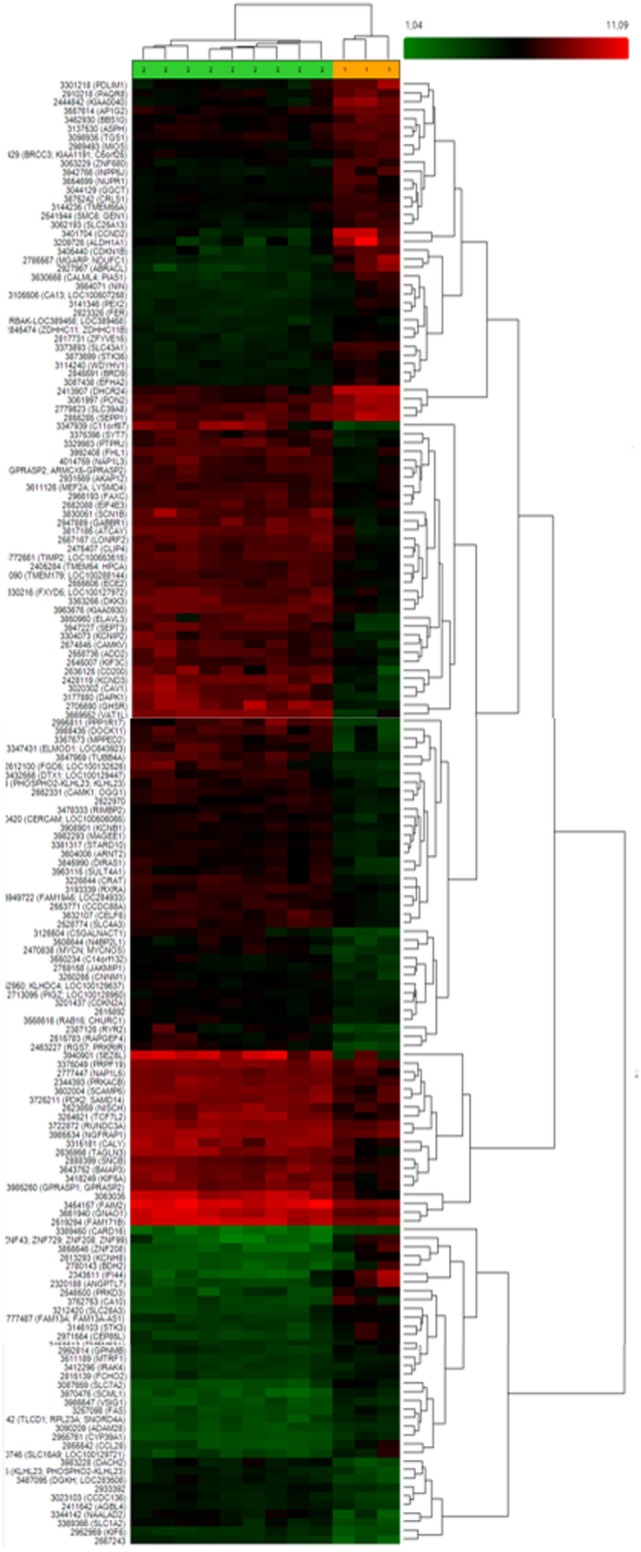
**Heat map and hierarchical clustering of 168 differently expressed genes between invasive and non-invasive corticotrophinomas showing that these groups exhibit a distinct gene expression profile**. Different genes are represented in different rows and different experiments in different columns. The colored bar above the heat map (horizontal dimension) indicates the grouping variable—green for non-invasive (*n* = 9) and orange for invasive tumors (*n* = 3). The normalized expression value of each gene is color coded, from red for higher expressions to green for lower expressions and genes with no change in expression are represented in black.

After that, we applied a false discovery rate (FDR) cutoff of 0.05 to obtain a robust list of 168 differentially expressed genes (Table 5; Table S2 in Supplementary Material; Figure 1A), in which downregulation was the most prevalent feature (*n* = 150). A heat map and hierarchical cluster of these 168 genes clearly demonstrated a different gene expression signature between invasive and non-invasive groups (Figure 2).

**Table 5:** **Differentially expressed genes in invasive versus non-invasive corticotrophinomas**.

Gene symbol	Fold change	Gene symbol	Fold change	Gene symbol	Fold change	Gene symbol	Fold change
*IFI44*	43.02	*GPNMB*	2.76	*JAKMIP1*	−2.29	*ADD2*	−3.54
*ZNF676*	29.28	*VSIG1*	2.76	*JPH3*	−2.29	*TCF7L2*	−3.59
*CCND2*	27.61	*SLC39A8*	2.75	*FAXC*	−2.31	*MYCN*	−3.73
*ANGPTL7*	15.2	*IRAK4*	2.59	*CCDC88A*	−2.33	*C14orf132*	−3.94
*ZNF208*	13.55	*BBS10*	2.58	*CERCAM*	−2.33	*GNAO1*	−3.95
*ABRACL*	11.87	*TMEM63A*	2.52	*NAP1L5*	−2.36	*DACH2*	−4.04
*ALDH1A1*	10.95	*TLCD1*	2.51	*PRPF19*	−2.39	*LONRF2*	−4.05
*KCNH8*	10.36	*TMEM55A*	2.34	*CLIP4*	−2.45	*FXYD5*	−4.09
*MGARP*	8.7	*MIOS*	2.33	*CAMK1*	−2.46	*CAMKV*	−4.1
*SLC7A2*	7.35	*CRLS1*	2.28	*ID 2622970*	−2.49	*SULT4A1*	−4.16
*KIAA0040*	7.11	*FER*	2.26	*KIF5A*	−2.5	*KCNIP2*	−4.26
*CA10*	7.06	*ASPH*	2.23	*MAGEE1*	−2.51	*DKK3*	−4.28
*DHCR24*	6.87	*EFHA2*	2.22	*RXRA*	−2.57	*PTPRJ*	−4.29
*PRKD3*	6.81	*ZFYVE16*	2.2	*ARNT2*	−2.61	*SCN1B*	−4.29
*CCL28*	6.43	*BRD9*	2.19	*BAIAP3*	−2.62	*CALY*	−4.33
*CDKN1B*	5.87	*MTRF1*	2.19	*AKAP12*	−2.68	*CD200*	−4.56
*BDH2*	5.8	*WDYHV1*	2.17	*SCAMP5*	−2.69	*DTX1*	−4.66
*CEP85L*	5.77	*ZDHHC11*	2.16	*SLC4A3*	−2.69	*ID 2667243*	−4.67
*CALML4*	5.55	*SMC6*	2.15	*DGKH*	−2.7	*SLC1A2*	−4.88
*SCML1*	5.33	*ADAM28*	2.1	*CCDC136*	−2.71	*DIRAS1*	−4.97
*SLC28A3*	5.11	*RBAK*	2.06	*RIMBP2*	−2.74	*RYR2*	−5.5
*STK3*	4.78	*BRCC3*	2.05	*CNNM1*	−2.75	*TUBB4A*	−5.52
*FAM13A*	4.25	*TGS1*	2.05	*GABBR1*	−2.76	*ELMOD1*	−5.75
*INPP5J*	4.24	*GGCT*	2.04	*STARD10*	−2.79	*FGD5*	−6.16
*PDLIM1*	4.14	*CDKN2A*	−2.01	*EIF4E3*	−2.8	*TAGLN3*	−6.39
*SLC43A1*	4.14	*PRKACB*	−2.02	*TMEM54*	−2.81	*RGS7*	−6.48
*ZNF680*	3.79	*ID 615892*	−2.07	*KLHL23*	−2.94	*DOCK11*	−7.17
*PON2*	3.71	*KCNB1*	−2.08	*N4BP2L1*	−2.95	*ATCAY*	−7.18
*NUPR1*	3.63	*KIF3C*	−2.09	*PHOSPHO2-KLHL23*	−2.96	*KCND3*	−7.65
*PEX2*	3.57	*PIGZ*	−2.1	*ECE2*	−3.01	*DAPK1*	−7.8
*SEPP1*	3.36	*NGFRAP1*	−2.11	*NISCH*	−3.03	*CSGALNACT1*	−8.2
*NIN*	3.23	*MEF2A*	−2.12	*SYT7*	−3.03	*CAV1*	−9.03
*CARD16*	3.11	*FAM19A5*	−2.14	*CRAT*	−3.04	*NAALAD2*	−9.34
*PAQR8*	3.05	*RAB15*	−2.14	*GPRASP1*	−3.04	*MPPED2*	−9.51
*FCHO2*	3.02	*RUNDC3A*	−2.15	*KIAA0930*	−3.04	*ELAVL3*	−9.7
*SLC25A13*	3.02	*TMEM179*	−2.17	*NAP1L3*	−3.06	*GHSR*	−10.13
*CYP39A1*	3	*PDK2*	−2.2	*TIMP2*	−3.07	*ID 3063035*	−10.29
*SLC16A9*	2.96	*KIF6*	−2.23	*GPRASP2*	−3.26	*PPP1R17*	−10.81
*STK35*	2.93	*CELF6*	−2.26	*FAM171B*	−3.29	*SEPT3*	−11.83
*FAS*	2.91	*SNCB*	−2.26	*FAIM2*	−3.3	*C11orf87*	−19.63
*AP1G2*	2.89	*AGBL4*	−2.27	*FHL1*	−3.33	*VAT1L*	−23.83
*CA13*	2.82	*ID 933392*	−2.28	*RAPGEF4*	−3.33	*SEZ6L*	−49.33

*ANOVA p-value <0.05; fold change cut off = 2; false discovery rate <0.05; ID, transcript ID, no gene symbol available*.

Figure 1B demonstrates the transcriptomic data between invasive and non-invasive corticotrophinomas, indicating some genes that will be properly discussed later in this paper. Some of the differentially expressed genes have known functions in cancer, cell cycle, and death (overexpressed: *CCND2*; underexpressed: *DAPK1, CDKN2A*), transcription factor, and gene expression regulation (overexpressed: ZNF676), cellular homeostasis, adhesion, and motility (overexpressed: *KCNH8, DHCR24, MGARP, PRKD3*; underexpressed: *DOCK11, SPON1, SEPT3*), and protein binding (underexpressed: *TIMP2*).

A list of 20 most significantly and functionally relevant genes differentially expressed between these groups is shown in Table 6.

**Table 6:** **Twenty most significantly over- or underexpressed genes in invasive versus non-invasive corticotrophinoma groups**.

Gene symbol	Description	Fold change (linear)	ANOVA	FDR

			*p*-Value	*p*-Value
*IFI44*	Interferon-induced protein 44	43.02	0.00012	0.025879
*CCND2*	Cyclin D2	27.61	0.00003	0.013711
*ZNF676*	Zinc-finger protein 676	29.28	0.000198	0.030477
*ANGPTL7*	Angiopoietin-like 7	15.20	0.000035	0.015151
*KCNH8*	Potassium voltage-gated channel member 8	10.36	0.000554	0.045037
*MGARP*	NADH dehydrogenase (ubiquinone) 1	8.70	0.00004	0.015427
*DHCR24*	24-dehydrocholesterol reductase	6.87	7.97E−07	0.004751
*PRKD3*	Protein kinase D3	6.81	0.000105	0.024751
*CEP85L*	Centrosomal protein 85kda-like	5.77	0.000026	0.013222
*SPON1*	Spondin 1. Extracellular matrix protein	−16.79	0.011393	0.16522
*SEPT3*	Septin 3	−11.83	0.00009	0.022919
*MPPED2*	Metallophosphoesterase domain-containing 2	−9.51	0.000086	0.022798
*CSGALNACT1*	Chondroitin sulfate *N*-acetylgalactosaminyltransferase 1	−8.20	0.000234	0.031613
*DAPK1*	Death-associated protein kinase 1	−7.80	0.000037	0.015151
*DOCK11*	Dedicator of cytokinesis 11	−7.17	0.000299	0.034763
*RGS7*	Regulator of G protein signaling 7	−6.48	0.00001	0.010564
*PEX2*	Peroxisomal biogenesis factor 2	−3.57	0.0006	0.046209
*TIMP2*	TIMP metallopeptidase inhibitor 2	−3.07	0.000178	0.029718
*ARNT2*	Aryl-hydrocarbon receptor nuclear translocator 2	−2.61	0.000011	0.011139
*CDKN2A*	Cyclin-dependent kinase inhibitor 2A	−2.01	0.000344	0.035806

*FDR, false discovery rate*.

Further, to analyze the biological significance of these genes, we used DAVID and Enrich chip annotation tools to reveal the functional description, classification, and location of the differentially expressed genes. The annotation results showed that of the 168 differentially expressed genes, 92 were known genes associated with a diverse set of biological pathways. Among these pathways, nine showed altered expression of at least three genes (Table 7). They include the TGF-β and G protein signaling pathways, DNA damage response pathway, and pathways associated with focal adhesion.

**Table 7:** **List of signaling pathways affected with at least three downregulated and/or upregulated genes**.

Pathway	#Total	Downregulated	Upregulated
Vitamin D receptor pathway	5	*RXRA, CD200, TIMP2, CDKN2A*	*CDKN1B*
TGF beta signaling pathway	4	*CAV1, MEF2A, PIAS1*	*ZFYVE16*
G protein signaling pathways	4	*GNAO1, PRKACB, AKAP12*	*PRKD3*
DNA damage response (only ATM dependent)	3	*CDKN2A*	*CDKN1B, CCND2*
DNA damage response	3	–	*FAS, CDKN1B, CCND2*
G1 to S cell cycle control	3	*CDKN2A*	*CCND2, CDKN1B*
Focal adhesion	3	*CAV1*	*SEPP1, CCND2*
Nuclear receptors meta-pathway	3	*RXRA*	*CDKN1B, SLC39A8*
miRNA regulation of DNA damage response	3	–	*FAS, CDKN1B, CCND2*

### Quantitative Gene Expression Analysis

**Figure 3:**
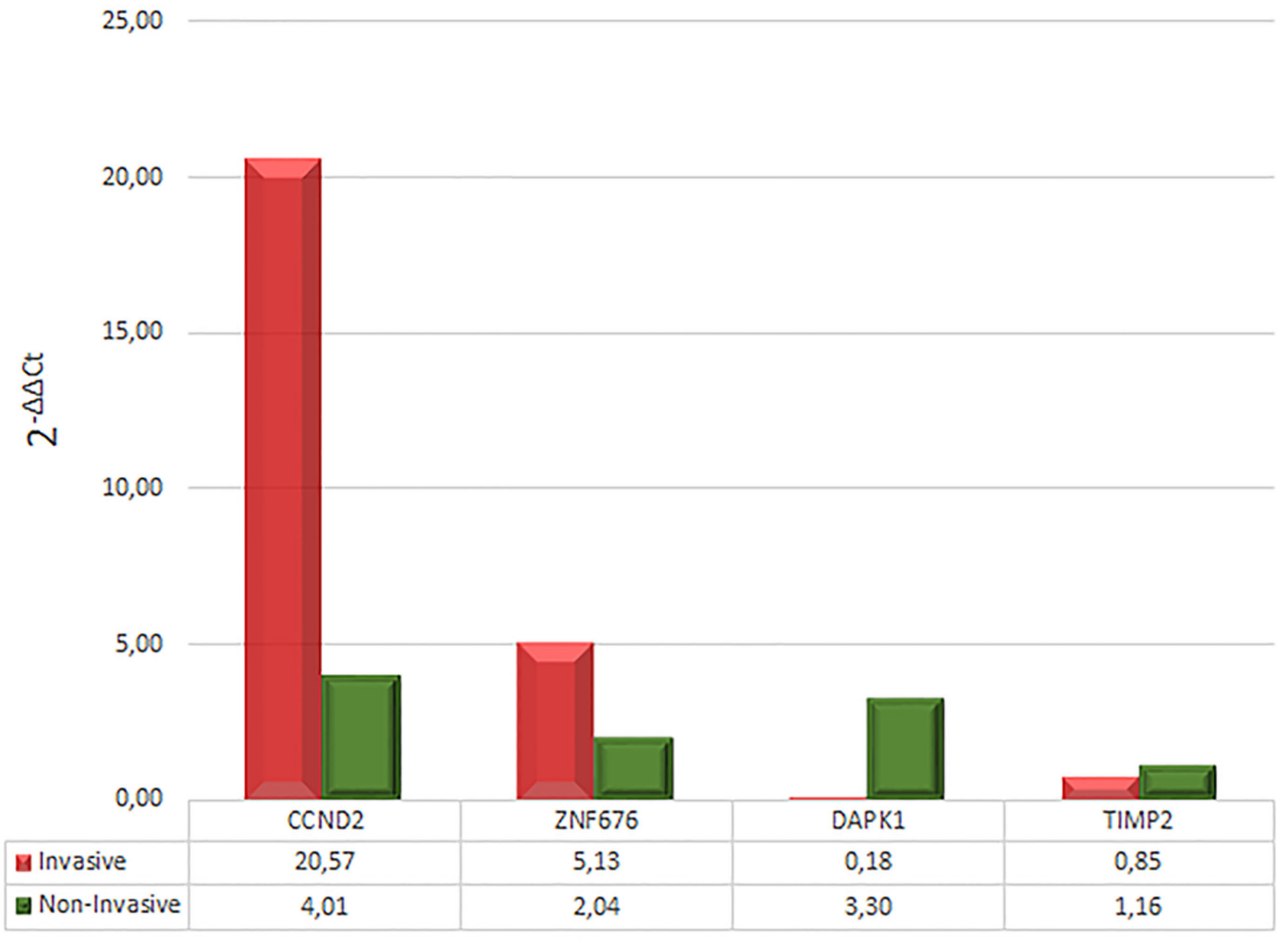
**Relative expression levels (fold change) of *CCND2, ZNF676, DAPK1*, and *TIMP2***.

Based on the degree of over- or underexpression in invasive versus non-invasive corticotrophinomas, expression of four genes was quantified by qRT-PCR. This analysis confirmed the overexpression of *CCND2* (mean increase of 20.57-fold in invasive and 4.01-fold in non-invasive, in relation to calibrator) and *ZNF676* (mean increase of 5.13-fold in invasive and 2.04-fold in non-invasive, in relation to calibrator), and the underexpression of *DAPK1* (mean increase of 0.18-fold in invasive and 3.3-fold in non-invasive, in relation to calibrator). Regarding to *TIMP2*, it was possible to observe only a tendency to underexpression (mean increase of 0.85 times in invasive and 1.16 times in non-invasive, in relation to calibrator) (Figure 3).

Regarding to somatic *USP8* mutations, they were identified in 5 (non-invasive corticotrophinomas) out of 18 patients (27.7%) of validation cohort.

## Discussion

In the microarray study cohort, we identified six *USP8* mutations in 12 samples (50%). In the patients included in the validation study, we could identify somatic *USP8* mutations in 5 (non-invasive corticotrophinomas) out of 18 patients (27.7%). According to Reincke et al. (17) and Perez-Rivas et al. (18), these mutations occur in ≈36% of patients with CD. Interestingly, the presence of *USP8* mutations did not interfere in the transcriptome expression analysis results comparing invasive vs. non-invasive tumors and in its validation study.

Our study design was original as we compared the gene expression profile of MIC and MAC, also considering tumoral invasiveness. Previous microarray studies have identified genes differentially expressed in pituitary adenomas including corticotrophinomas, but without taking into account their tumor size classification and/or invasiveness (33–35).

Despite our analysis did not show a clear functional distinction between MAC and MIC groups, we were able to identify genes selectively over- and underexpressed in the MAC invasive group, which exhibited a distinct gene expression signature from MIC and non-invasive MAC corticotrophinomas. Among the most differentially expressed genes, we highlighted *CCND2* (*cyclin D2*) and *ZNF676* (*zinc-finger protein 676)*, which were increased by 27- and 29-fold, respectively, *DAPK1* and *TIMP2* which was decreased by 8-fold and 3-fold, respectively.

*CCND2* is a crucial cell cycle-regulatory protein; its overexpression is described in several human neoplasms, including colorectal adenomas and gastric cancer, and it is associated with a poor prognosis in gastric cancer (36). Genetic aberrations of *CCND2* are also frequently described in gliomas and hematologic malignancies (37, 38).

The ZNF676 is a transcriptional regulator with an interesting suggested role in telomere homeostasis in humans (39). Telomere dysfunction is a common cause and a hallmark of cancer that can lead to genomic instability when associated with loss of cell cycle control (40). It is unclear how ZNF676 controls the length of telomeres. Theoretically, it can modify the telomere length (a) by directly binding to DNA, and it might alter the expression (repression/activation) of genes engaged in telomere maintenance and (b) by binding specifically to and stabilizing the G-quadruplex structure of telomeric DNA (39–42). However, it was recently demonstrated that pituitary tumors do not exhibit alterations in telomeric length, suggesting that telomere biology does not play an important role in pituitary tumor development (43).

DAPK1 is a positive mediator of gamma-interferon-induced programmed cell death with a putative role of metastasis suppressor. *DAPK1* expression silencing due to promoter methylation has been frequently found in lung cancer, in which cells with lack of *DAPK1* expression appear to be more invasive and more metastatic (41). This gene was also found to be frequently overmethylated in head and neck cancers and in immunodeficiency-related lymphomas (44–46).

TIMP2 is a tissue inhibitor of the matrix metalloproteinase family (MMP) and has been studied in several human tumors, in which a negative correlation between *TIMP2* expression and aggressiveness/malignancy was demonstrated (47). Recently, the predictive roles of *MMP9* and *TIMP1* and 2 in the invasiveness of prolactinomas were studied, and higher *MMP9* expression and underexpression of *TIMP2* were found in invasive tumors (48). Therefore, it is possible that *TIMP2* could also be a potential marker of invasion in corticotrophinomas.

Among our differentially expressed genes, some corroborated published studies that compared gene expression of normal pituitary tissue and pituitary adenomas: the overexpression of *CCND1* and underexpression of *CDKN2A*. *CCND1* encodes the cyclin D1 protein, which together with other cyclins, acts in the regulation of cyclin-dependent kinases (CDKs). The activation or inactivation of kinases mechanisms is often associated with cell cycle (49). Additionally, the overexpression of *CCND1* is known to be present in many neoplasms, malignant, and non-malignant, and it is considered one of the most important tumorigenic factors (19, 50). Despite this, few studies investigated its role in pituitary adenomas, only two studies have observed the overexpression of *CCND1* in adenomas compared with normal pituitary tissue. In addition, the increased expression of cyclin D1 was associated with a greater recurrence of the disease (50, 51). The *CDKN2A* also encodes a CDK inhibitor, p16, directly involved in cell cycle control (52). It has been demonstrated that *CDKN2A* methylation occurs in the entire locus, in all subtypes and pituitary tumors. In addition, the dysfunction of p16 was associated with the increased size of these tumors (53). Another study went further and demonstrated that functional corticotrophinomas exhibited an expression of this gene up to four times higher than non-functioning adenomas. The authors suggested that this result could explain why functional corticotrophinomas tend to be smaller than other types of adenomas (54). Corroborating these correlations, in our cohort of invasive corticotrophinomas, with underexpression of *CDKN2A*, the mean tumor size was significantly higher (27.7 ± 11.2 mm) than in the non-invasive corticotrophinomas (10.9 ± 4.8 mm), even when we consider only the size of MACs (15.40 ± 4.04 mm).

Interestingly, in our study, both the pituitary tumor-transforming gene 1 (*hPTTG1*) and *VEGF* were found to be underexpressed in the invasive group. *hPTTG1* encodes a mammalian securin found to be overexpressed in several tumors and to transform cells *in vitro* and *in vivo*, and VEGF is the most frequently studied angiogenic factor that is involved in endothelial cell proliferation, vascular permeability, and cell motility (8, 20). It has been reported *hPTTG1* overexpression in pituitary adenoma (21, 22) and its positive correlation with invasiveness (23, 55). As these studies used different methodologies and they included non-corticotroph pituitary adenoma, it might explain the reason for the discrepancy in our results.

In a similar manner, *FGFR4* did not show significant expression difference between invasive and non-invasive corticotrophinomas. It is important to notice that *hPTTG1* and *FGFR4* overexpression was previously positively correlated with elevated Ki-67 nuclear proliferation index in pituitary adenomas (>3%) (12, 23). However, in our study Ki-67 did not indicate aggressiveness; therefore, this might contribute to the difference obtained in microarray expression results.

Likewise, *CDN1B* that encodes p27^kip1^ protein, member of the Cip/Kip family of CDK inhibitors, was overexpressed in our cohort of invasive corticotrophinomas, although it has been demonstrated that the loss of expression of *CDN1B* may result in pituitary hyperplasia and tumorigenesis (19). Therefore, the reason for this discrepancy of our result with the previous published is not clear.

It is noteworthy to mention that, in the presence of the overexpression of *CCND2* and *ZNF676*, and underexpression of *DAKP1* and *TIMP2*, patients from the invasive group demonstrated a higher mean of presurgical ACTH (102.3 ± 52.2 pg/mL, normal range <46 pg/mL) compared to patients from the non-invasive group (51.7 ± 15.9 pg/mL, normal range 50–310 μg/24 h). In contrast, patients harboring non-invasive corticotrophinomas presented higher concentrations of urinary cortisol (639.6 ± 358.0 μg/24 h) when compared to patients harboring invasive corticotrophinomas (406.0 ± 34.8 μg/24 h).

Despite the intrinsic difficulty of handling and obtaining viable corticotroph tumor tissue for molecular studies and the low incidence of invasive corticotrophinomas, we were able to use a larger cohort of new patients and perform qRT-PCR to the highlighted genes. Our validation results corroborated the initial findings of the microarray study, as we observed *CCND2* and *ZNF676* overexpression and *DAPK1* and *TIMP2* underexpression.

Since there are no other molecular studies available comparing invasive and non-invasive corticotrophinomas, this study is an important contribution to the investigation of the biological behavior of these tumors.

## Conclusion

We identified a differential pattern of genetic signature in a subgroup of MACs, supporting a genetic influence on the pathogenesis of corticotrophinomas. This study highlighted genes that might contribute for the improvement of molecular diagnosis of invasive corticotrophinomas. Additional analysis are necessary to evaluate the differential protein expression in a larger cohort, therefore they could be used in clinical practice.

## Ethical Standards

The authors declare that the experiments comply with the current laws of their country.

## Author Contributions

LA: PhD student responsible for all experiments, biostatistical analysis and interpretation, as well as manuscript writing; AL: biostatistical analysis advisor; MC and CM: provided with tumor samples and patient data for validation cohort and manuscript reviewer; MB and MM: manuscript reviewers; ET: technical/experimental advisor, statistical analysis and interpretation and manuscript reviewer; MF: project mentor and advisor, responsible for patient clinical management and manuscript reviewer.

## Conflict of Interest Statement

The authors declare that the research was conducted in the absence of any commercial or financial relationships that could be construed as a potential conflict of interest.

## Footnotes

^1^https://david.ncifcrf.gov/home.jsp.

^2^http://amp.pharm.mssm.edu/Enrichr/.

## Supplementary Material

Figure S1 **Electrophoresis on agarose 1% gel for visualization of the PCR products of the genes *GAPDH, TPIT*, and *POU1FT***. The expression of *GAPDH* was used as an endogenous control. Columns 1–15 represent our samples, and column 16 is the negative control. Samples 11, 12, and 15 were excluded because they had visibly positive amplification of *POU1FT*, suggesting contamination with non-corticotrophic tissue.
