# Corrigendum: Transcriptome Analysis Showed a Differential Signature Between Invasive and Non-invasive Corticotrophinomas

**DOI:** 10.3389/fendo.2019.00567

**Published:** 2019-08-22

**Authors:** Leonardo Jose Tadeu de Araújo, Antonio Marcondes Lerario, Margaret de Castro, Clarissa Silva Martins, Marcello Delano Bronstein, Marcio Carlos Machado, Ericka Barbosa Trarbach, Maria Candida Barisson Villares Fragoso

**Affiliations:** ^1^Laboratory of Hormones and Molecular Genetics LIM-42, University of São Paulo Medical School, São Paulo, Brazil; ^2^Laboratory of Quantitative Pathology, Center of Pathology, Adolfo Lutz Institute, São Paulo, Brazil; ^3^Division of Metabolism, Endocrinology and Diabetes, University of Michigan, Ann Arbor, MI, United States; ^4^Internal Medicine Department, Ribeirao Preto Medical School, University of São Paulo, Ribeirao Preto, Brazil; ^5^Neuroendocrine Unit, Division of Endocrinology and Metabolism, University of São Paulo Medical School, São Paulo, Brazil; ^6^Endocrinology Service, AC Cancer Center, São Paulo, Brazil; ^7^Laboratory of Cellular and Molecular Endocrinology LIM-25, University of São Paulo Medical School, São Paulo, Brazil

**Keywords:** Cushing's disease, gene expression, neuroendocrine tumors, microarray, anterior pituitary

In the original article, there was an error. We stated that *USP8* genetic abnormalities were not identified in DNA from tumor samples analyzed by microarray technology.

A correction has been made to **Abstract, Paragraph Number 1, Lines 18–19**.

Somatic mutations in *USP8* were also investigated and mutations were identified in six cases.

A correction has been made to **Results, Paragraph Number 1**.

Before microarray analysis, we performed the screening for mutations in *USP8* in our patients, and somatic variants were found in patients #2 and #5 (p.Ser718Pro), #4 (p.Ser718Cys), patients #3 and #6 (p.Pro720Arg), and #9 (p.Pro720Gln). Both mutations were found in heterozygosis and have been previously described (17, 18).

A correction has been made to **Discussion, Paragraph Number 1**.

In the microarray study cohort, we identified six *USP8* mutations in 12 samples (50%). In the patients included in the validation study, we could identify somatic *USP8* mutations in 5 (non-invasive corticotrophinomas) out of 18 patients (27.7%). According to Reincke et al. (17) and Perez-Rivas et al. (18), these mutations occur in ≈36% of patients with CD. Interestingly, the presence of *USP8* mutations did not interfere in the transcriptome expression analysis results comparing invasive vs. non-invasive tumors and in its validation study.

The authors apologize for this error and state that this does not change the scientific conclusions of the article in any way. The original article has been updated.

