# Supplementary material for: Transcriptome Analysis Showed a Differential Signature between Invasive and Non-invasive Corticotrophinomas
Source: Front Endocrinol (Lausanne). 2017 Mar 22;8:55. doi: 10.3389/fendo.2017.00055 (PMC5360720; doi:10.3389/fendo.2017.00055)
Supplement: Supplementary file 1 [file Table_1.DOC]

| **Orientation** | **Function*** | **Sequence** |
| --- | --- | --- |
| Forward | PCR | 5'-GCAGAATACTTTGGAGTGATTTCTT-3' |
| Reverse | PCR | 5'-TCCAACTCCCTGACACTAACA-3' |
| Forward | PCR | 5'-CTTGACCCAATCACTGGAAC-3' |
| Reverse | PCR | 5'-CAGCACATTATTTTAGTTCTAGGAGTT-3' |
| Forward | SDM DEL1 | 5'-CTGAAGCGCTCCTACTCCTCTATAACCCAGGCTATTCAAG-3' |
| Reverse | SDM DEL1 | 5'-CTTGAATAGCCTGGGTTATAGAGGAGTAGGAGCGCTTCAG-3' |
| Forward | SDM DEL2 | 5'-CCAAACTGAAGCGCTCCTACTCAGATATAACCCAGGCTATTC-3' |
| Reverse | SDM DEL2 | 5'-GAATAGCCTGGGTTATATCTGAGTAGGAGCGCTTCAGTTTGG-3' |
| Forward | SDM DEL3 | 5'-CAAACTGAAGCGCTCCTACTCGGCTATTCAAGAGGAAGAG-3' |
| Reverse | SDM DEL3 | 5'-CTCTTCCTCTTGAATAGCCGAGTAGGAGCGCTTCAGTTTG-3' |
| Forward | SDM DEL4 | 5'-CCAAACTGAAGCGCTCCTACTCCCAGGCTATTCAAGAGGAAGAG-3' |
| Reverse | SDM DEL4 | 5'-CTCTTCCTCTTGAATAGCCTGGGAGTAGGAGCGCTTCAGTTTGG-3' |
| Forward | SDM P720Q | 5'-GCTCCTACTCCTCCCAAGATATAACCCAG-3' |
| Reverse | SDM P720Q | 5'-CTGGGTTATATCTTGGGAGGAGTAGGAGC-3' |

**Supplemental Table 1.** List of primers used for amplification and site-directed mutagenesis of *USP8*.

*PCR: Amplification by polymerase chain reaction; SDM: Site-directed mutagenesis.
