# Supplementary figures and images for: Transcriptome Analysis Showed a Differential Signature between Invasive and Non-invasive Corticotrophinomas
Source: Front Endocrinol (Lausanne). 2017 Mar 22;8:55. doi: 10.3389/fendo.2017.00055 (PMC5360720; doi:10.3389/fendo.2017.00055)

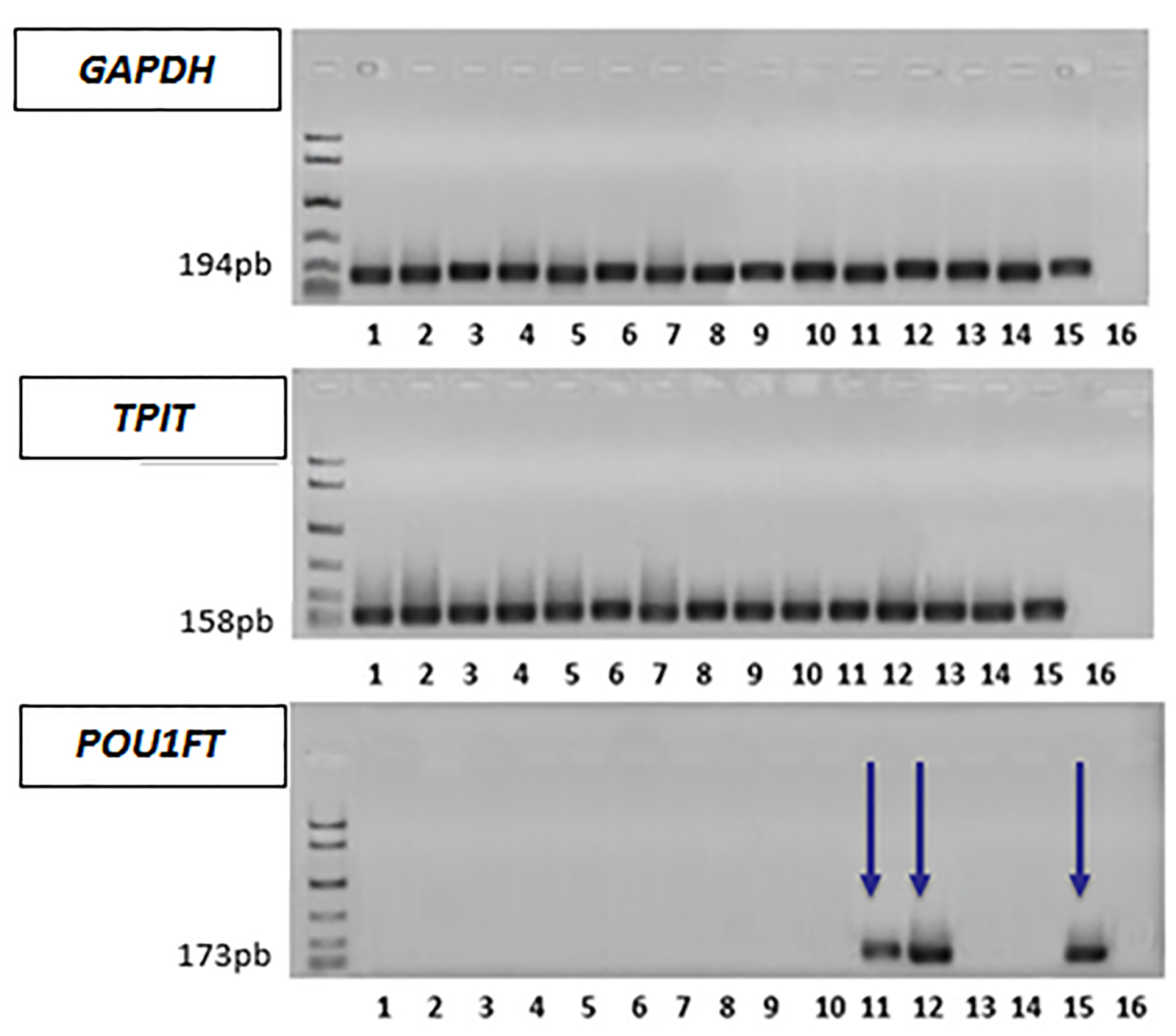

Supplement: Figure S1 — Electrophoresis on agarose 1% gel for visualization of the PCR products of the genes GAPDH, TPIT, and POU1FT. The expression of GAPDH was used as an endogenous control. Columns 1–15 represent our samples, and column 16 is the negative control. Samples 11, 12, and 15 were excluded because they had visibly positive amplification of POU1FT, suggesting contamination with non-corticotrophic tissue. [file Image_1.TIF]
